# Synthetic fossilization of soft biological tissues and their shape-preserving transformation into silica or electron-conductive replicas

**DOI:** 10.1038/ncomms6665

**Published:** 2014-12-08

**Authors:** Jason L. Townson, Yu-Shen Lin, Stanley S. Chou, Yasmine H. Awad, Eric N. Coker, C. Jeffrey Brinker, Bryan Kaehr

**Affiliations:** 1 Division of Molecular Medicine, Department of Internal Medicine, The University of New Mexico, Albuquerque, New Mexico 87131, USA; 2 Center for Micro-Engineered Materials, The University of New Mexico, Albuquerque, New Mexico 87131, USA; 3 Advanced Materials Laboratory, Sandia National Laboratories, Albuquerque, New Mexico 87185, USA; 4 Department of Chemical and Biological Engineering, The University of New Mexico, Albuquerque, New Mexico 87131, USA

## Abstract

Structural preservation of complex biological systems from the subcellular to whole organism level in robust forms, enabling dissection and imaging while preserving 3D context, represents an enduring grand challenge in biology. Here we report a simple immersion method for structurally preserving intact organisms via conformal stabilization within silica. This self-limiting process, which we refer to as silica bioreplication, occurs by condensation of water-soluble silicic acid proximally to biomolecular interfaces throughout the organism. Conformal nanoscopic silicification of all biomolecular features imparts structural rigidity enabling the preservation of shape and nano-to-macroscale dimensional features upon drying to form a biocomposite and further high temperature oxidative calcination to form silica replicas or reductive pyrolysis to form electrically conductive carbon replicas of complete organisms. The simplicity and generalizability of this approach should facilitate efforts in biological preservation and analysis and could enable the development of new classes of biomimetic composite materials.

## Abstract (web summary)

Imaging biological tissues has long been an issue, particularly with regard to manipulation and dissection for SEM. Here, the authors present a simple technique for the stabilization of biological tissues via a synthetic fossilization process, requiring minimal expertise or equipment and involving few steps.

## Graphical abstract

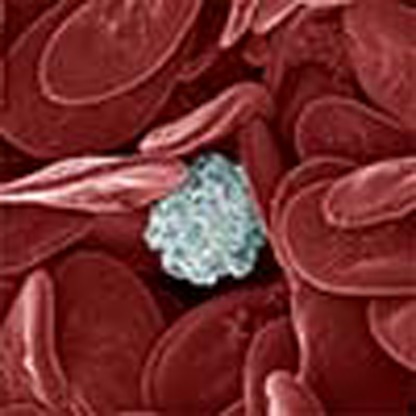

The invention of electron microscopy (EM) in the early 20th century broadly impacted the physical and materials sciences, but proved particularly important for biological research by enabling macromolecular complexes, ultrastructures and other optically unresolvable features of cells and tissues to be studied in context^1^,^2^. Throughout the technique’s history, EM instrumentation has steadily improved allowing for increasingly better resolution and imaging in both scanning electron (SEM) and transmission electron microscopy (TEM; for example, aberration correction^3^), and providing procedures that enable *in situ* imaging of dynamic processes^4^ and hydrated samples^5^, as well as other techniques for reconstructing three-dimensional (3D) images (for example, cryo-tomography^6^ and serial block-face SEM^7^). Yet, despite consistent advancements in instrumentation, techniques for the preparation of biological materials for EM remain largely unchanged since first put into practice^2^. These often elaborate procedures designed to preserve delicate biological samples under the harsh conditions of EM (high-vacuum and energy flux) remain cumbersome to the non-specialist and thus limit their broader usage. For example, methods such as cryofixation (vitrification) currently come closest to capturing the most ‘accurate’, native state of a biomolecular structure because of the rapid stabilization of the specimen within vitreous ice through cold plunge, but, in particular for macroscopic tissues or organisms, it requires specialized equipment, subsequent processing (for example, replication, fracture and/or cryo-focused-ion beam; cryo-FIB) and considerable expertise^2^. Freeze fracture, though an important development for elucidating cellular ultrastructure, remains a highly specialized technique largely because of its overall complexity and multitude of intricate steps^8^. Chemical fixation using aldehyde cross-linkers is a relatively simple approach, but dehydration via replacement of water with sequential, increasing concentrations of low viscosity, high vapour pressure solvents (for example, ethanol, methanol, acetone) and subsequent drying necessary for imaging in vacuum can degrade sample architectures via de-wetting and other drying stresses, which can be significant for small features^9^. Alternatively, secondary and tertiary fixation is employed using osmium tetroxide followed by uranyl acetate and subsequent dehydration using critical point or freeze drying^5^, but this route substantially increases both process complexity and use of toxic chemicals. Processing of specimens using embedding resins results in a moulded polymer block that enables thin sectioning for cross-sectional observation in TEM, serial block-face SEM or focused ion beam SEM (FIB/SEM)^10^, but this process destroys the 3D structure as a whole. Finally, for observation using SEM, biological specimens generally require coating with metal or carbon films to avoid charging. While these conductive coatings improve EM resolution, they have inherent granularity with grain sizes that can reach tens of nanometres as film thickness increases^11^; they can also alter topological features of soft materials and can render only exposed surfaces conductive. Uniform deposition on complex 3D structures is challenging because of shadowing effects using evaporated or sputtered metals.

To overcome limitations of existing methods of tissue/organism stabilization and imaging, the goal of this study was the development of a sample preparation procedure requiring few steps and minimal expertise or specialized equipment. We postulated a process that resulted in conformal structural stabilization from subcellular to organism scales, avoided embedding in polymer, and rendered an intrinsically conductive specimen resistant to high intensity energy and long-term degradation would provide new opportunities for biological analysis (for example, internal imaging, elemental contrast) and establish a new preparation method that complements the substantial recent developments in EM instrumentation. As a starting point, we considered natural mineralization processes that produce fossilized materials. Structural preservation of biological materials through fossilization requires an intricate alignment of optimum conditions that are achieved over long time scales by complex geological processes. Even if these are satisfied, preservation of soft tissue in natural fossils is extremely rare^12^. ‘Synthetic fossilization’ has been widely explored using, for example, wood, leaves, butterfly wings, pollen grains and diatoms as templates for material deposition and subsequent conversion^13^,^14^,^15^,^16^,^17^,^18^. However, these templates are already mechanically stable, comprising stiff polysaccharides (wood, butterfly wings) or bioinorganic composites (diatoms). Thus in contrast to soft tissues, they are intrinsically resistant to structural deformation upon drying and subsequent chemical processing. The extension of ‘synthetic fossilization’ to soft biomaterials under shape-preserving conditions would provide a new foundational approach for specimen preservation, create opportunities for conversion into more durable and EM-compatible materials, and serve as a facile approach to create new classes of biomimetic composite materials.

Here, we describe such an approach by which soft, biological tissues are replicated from the subcellular to the organismal scale in silica, a process we term silica bioreplication (SBR). We show shape and feature-preserving SBR of intact multicellular specimens (tissues derived from chicken embryos), inclusive of cells, extracellular matrices, tissues and organs as evidenced by generation of nearly identical inorganic (silica) replicas following removal of the biomolecular specimen template via high temperature calcination (500–600 °C) under oxidizing conditions. In addition, following SBR, specimens can be subjected to high temperature pyrolysis (800–1,000 °C) under reducing conditions to convert the organic constituents into conductive carbon. This procedure results in remarkable preservation of structure and enables whole, intact specimens to be imaged and sectioned *ad infinitum* and arbitrarily across the macro- to nanoscale without loss of resolution due to charging-induced specimen damage or imaging artifacts (as all internal and external features are carbonized and equally conductive). Images of cellular structure can be attained deep within tissue cavities of mechanically sectioned or FIBed organs. Finally, the resistance to damage of these specimens under high accelerating voltage and beam current provides excellent signal-to-noise ratios using backscattered electron (BSE) detection allowing, for example, single particle chemical imaging of injected gold nanoparticles (AuNPs) in a fracture plane of a chicken embryo liver.

## Results

### Silica bioreplication of chicken embryos

Recently, we observed that silicification of cultured mammalian cells derived from a range of tissues preserves cellular structure from the nano (DNA, organelles and so on) to whole cell (micrometre) level^19^. This ability to preserve intact cells with nanoscale fidelity laid the groundwork to examine SBR of complex multicellular systems in which cells organize to form diverse tissue types with distinct 3D architectures. To explore these possibilities, we used chicken embryos (*Gallus gallus domesticus*), which have been commonly used as exemplary *in vivo* models in the study of developmental biology, nanomedicine and other organism-scale processes^20^,^21^. As chicken embryos are primarily composed of soft tissue during the first 10 days of development and have well-formed internal organs by day 17, their use over 3–17 days of development allows us to demonstrate the efficacy of SBR for structural preservation of a wide spectrum of soft tissues and organs. First, embryos were removed from fertilized eggs at day 3 of development by cutting the egg shell and removing the intact embryo and membranes^21^. Following sufficient development (0–14 days of incubation at 37 °C and >65% relative humidity), embryos were euthanized and dissected or fixed whole in 3.7% formaldehyde in phosphate-buffered saline (PBS) for a minimum of 24 h. After fixation, embryos or individual organs were rinsed in PBS and then incubated for 7 days or more in acidic saline media (pH 3, 0.9% NaCl) containing silicic acid (Si(OH)_4_, 0.1 M) in a sealed container at 37 °C. Under these isotonic conditions, Si(OH)_4_ self-condensation into bulk silica (SiO_2_), which would obscure all structural detail, is minimized (formation of bulk gels would occur only after approximately 3 weeks of aging); instead, as we have observed using individual proteins and matrices^22^, as well as single cells^19^, condensation only occurs when catalysed by proximal biomolecular components—first mediated via hydrogen-bonded interactions with silica precursors—and subsequently catalysed amphoterically from the spectrum of acid and base moieties presented at the biomolecular surface^19^. This enables the self-limiting formation of a nanoscopic (4 to 10-nm thick) silica replica of all cellular-to-organism level features. Following incubation in the silicic acid solution, embryos were washed in H_2_O (pH 3), incubated in 1:1 H_2_O/methanol (20 min) and 100% methanol (20 min) and air dried.

**Figure 1:**
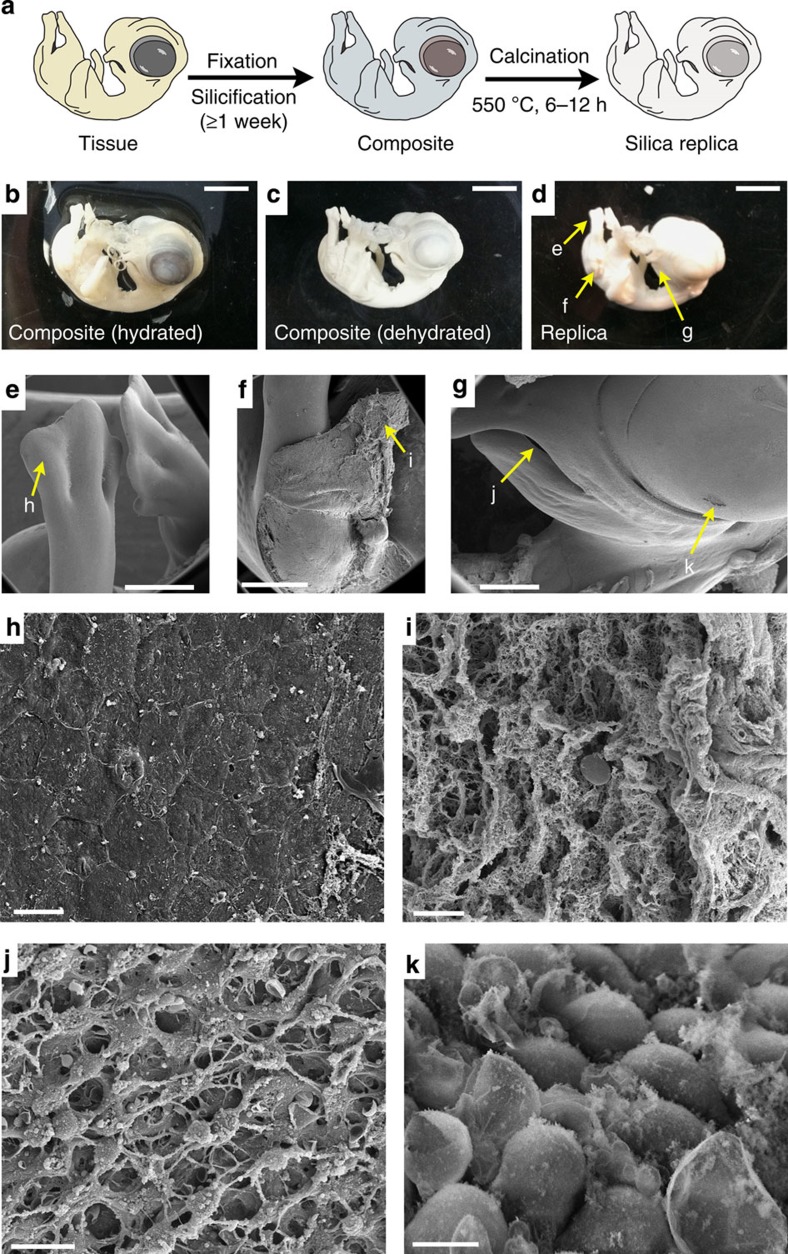
Silica bioreplication of a chicken embryo. (**a**) Schematic showing the silica bioreplication (SBR) process on an intact chicken embryo. The corresponding SBR composite of a 9-day-old chicken embryo (before (**b**) and after (**c**) dehydration) and (**d**) after calcination at 500 °C to produce a silica replica (scale bar (**b**–**d**), 5 mm). Embryo shows minimal shrinkage or shape change following dehydration (**c**) and calcination (**d**). (**e**–**g**) (Scale bars, 1 mm) Positions of the magnified images showing (**h**, scale bar, 10 μm) surface dermal tissue, (**i**, scale bar, 20 μm) subsurface cellular connective tissue, (**j**, scale bar, 10 μm) subsurface cells of the ocular membrane, (**k**, scale bar, 5 μm) surface cells of the developing tongue. SEM images were acquired following sputter coating of Au/Pd to a thickness of 10 nm.

The SBR procedure applied to an intact chicken embryo is shown schematically in Fig. 1a and detailed in a flow chart in Supplementary Fig. 1. Figure 1b,c show optical images of the resultant biocomposite specimen, both hydrated (that is, after silicification but before solvent washing and air drying) and dehydrated (following solvent washing and air drying), of a 9-day-old embryo.

### Organism-scale high fidelity silica replicas

To assess the extent and fidelity of silica deposition upon the template, the organic template was removed via calcination at 500 °C producing an inorganic silica replica (Fig. 1d). Magnified SEM images of surface and subsurface tissues (Fig. 1e–g and corresponding magnifications Fig. 1h–k) detail SBR over the entirety of the organism. Overall the images in Fig. 1 reveal the high fidelity replication afforded by SBR: over ~6 orders of magnification from the subcellular to organismal level and across diverse tissues types.

**Figure 2:**
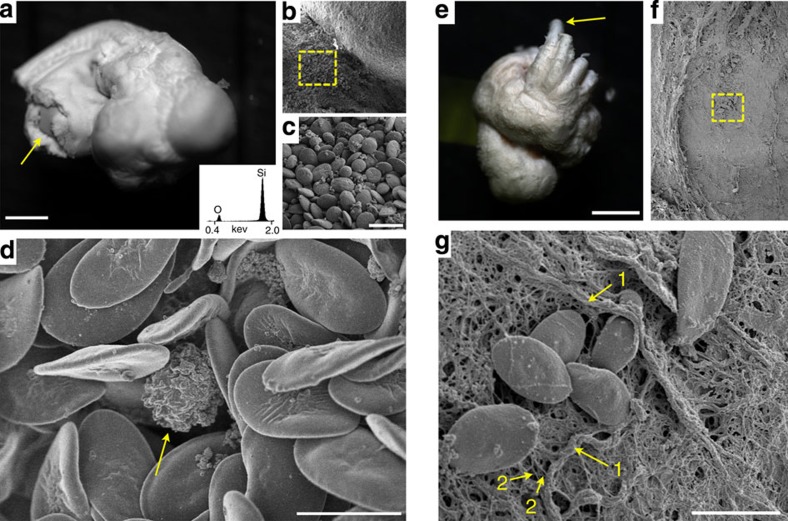
Structural preservation of deep tissue in silica replicas of chicken embryos. (**a**) Silica replica of a 4-day-old chicken embryo fractured post silicification and subsequently calcined (scale bar, 1 mm). The inset shows an energy-dispersive X-ray spectrum (EDS) of the silica replica. Arrow in **a** shows the exposed internal tissue magnified via SEM in **b** and further magnified in **c**, scale bar, 20 μm. (**d**) Silica replica of a white blood cell (arrow) nestled among SBR red blood cells within a blood vessel in chicken embryo liver (scale bar, 10 μm). (**e**) Silica replica of heart from a 17-day-old chicken embryo (scale bar, 2 mm). (**f**) SEM of the surface of a blood vessel (denoted by arrow in panel **e**) and further magnified in **g** showing red blood cell replicas bound to presumable elastin and collagenous fibres with diameters spanning microns (1) to 10s of nanometres (2, indicates ~60–80 nm fibres). SEM images were acquired following sputter coating of Au/Pd to a thickness of 10 nm.

Following verification of exterior surface structural preservation post silicification and calcination, we next examined the extent of silicification of internal organs and tissues. Figure 2a shows a calcined silica replica of a 4-day-old complete chicken embryo. We observed no substantial change in the overall dimensions of this embryo following high temperature treatment (500 °C) for 12 h, despite substantial weight loss (>50%) due to volatilization of the organic bulk biomolecular structure accompanied by continued condensation of silica, as indicated by thermogravimetric analysis (TGA) of SBR chicken embryo tissue under air (Supplementary Fig. 2). Indeed, in addition to the detailed surface features including vertebrae, developing brain, eyes and skin folds preserved in the silica embryo replica, Fig. 2a shows SEM images of the calcined silica replica of an embryo in which the interior of the embryo, inclusive of a lobe of the liver, has been exposed by fracture of the specimen along the midline. Closer examination at the fracture point (Fig. 2b) reveals preservation of complex structures from various tissue types, indicating silicification of tissues deep within the organism. As apparent in Fig. 2a–c, successive magnification of the indicated fracture point shows diverse cell types and extracellular matrix, including red blood cells and hepatocytes on the surface of the developing liver. Figure 2d shows a single white blood cell replica nestled among red blood cells located deep within a blood vessel of a calcined and fractured liver. SEM of the surface of large blood vessels on the silica heart replica (Fig. 2e–g) shows intact chicken red blood cell structures attached to replicated elastin and collagenous fibres (~10–150 nm) and fibre bundles (~400–1,000 nm). Although these results might be anticipated on the basis of our previous observation of individual cell replication^19^; the ready facile extension to a soft tissue—inclusive of all internal and external hierarchical structures—is remarkable given the fragility of unsupported tissues and organs in the absence of hydration. Organism-scale shape preservation combined with high fidelity nanoscale resolution of all extracellular and subcellular features within entire organs and throughout complete organisms indicates that SBR is macroscopically extensive, providing structural stability to soft tissue, yet nanoscopically thin. This is attributed to self-limiting silicic acid condensation at all biomolecular interfaces catalysed amphoterically by proximal membrane-associated proteins, carbohydrates (or other components). Occlusion of the catalytic biomolecular surface by silica naturally limits silica deposition to <10 nm, as was observed previously using single cell templates^19^, and the resultant SBR composite appears virtually indistinct from the biological specimen. Remarkably, the thin but extensive nanoscopic silica layer stabilizes the organism-scale features on drying and calcination to 500–600 °C despite substantial weight loss due to combustion of the organic template and further silica condensation. The stability of the SBR structure suggested opportunities for further material transformations. Thus we wondered whether subjecting SBR tissues to pyrolysis under inert atmosphere would yield a dimensionally preserved conductive replica via carbonization of the organic biological template. We reasoned that this transformation would produce a highly EM-compatible specimen (as all specimen surfaces should be intrinsically conductive) provided that the structure was preserved following high temperature treatment.

### Carbonization of silica composite specimens

**Figure 3:**
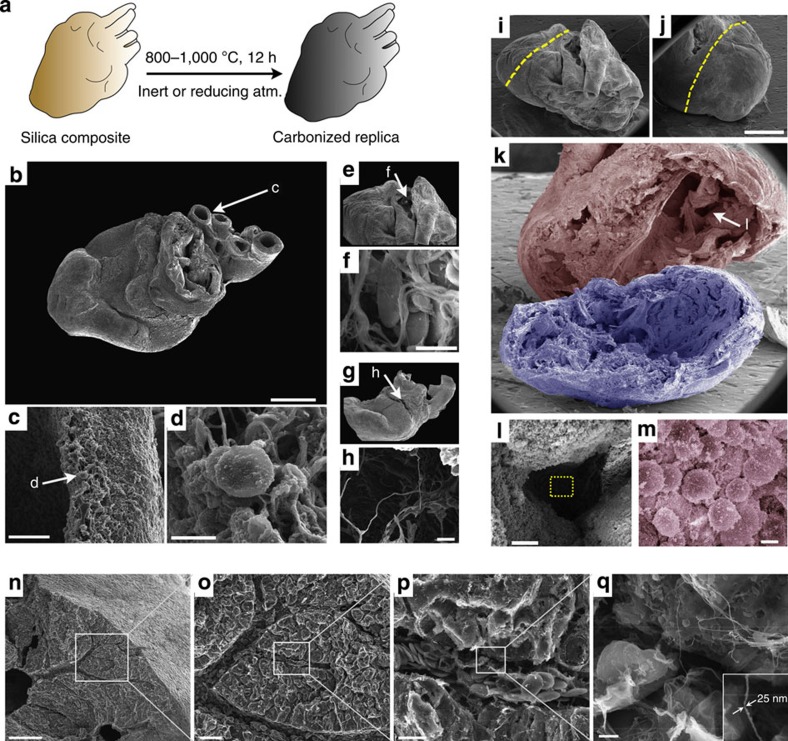
Shape-preserving conversion of silica bioreplication tissues into conductive specimens. (**a**) Schematic showing pyrolysis of a silicified heart into a carbonized silica replica. (**b**) SEM imaging (without conductive metal coating) of a heart carbonized at 1,000 °C (the background is subtracted for clarity; scale bar, 1 mm). (**c**,**d**) Show increasing magnification of an arterial wall (**c**, scale bar, 30 μm) and (**d**, scale bar, 5 μm) . (**e**) A crack in the top surface (indicated by arrow) reveals cellular and micro- (~1 μm) to nanoscale (~70 nm) extracellular fibres (**f**, scale bar, 5 μm). (**g**) A small opening in the side of the heart (indicated by arrow) reveals free-standing fibres and an interior chamber (**h**, scale bar, 10 μm). (**i**,**j**) Manual sectioning of a carbonized heart (dotted lines, **j**, scale bar, 1 mm) reveals the internal chambers (**k**). The two sections have been given false colour for clarity. Deep imaging into the area denoted by the arrow in **k** (**l**, scale bar, 50 μm) and further magnified (area in dashed rectangle in **l**) in **m** (scale bar, 5 μm) reveals surface bound cells ~1.5 mm within the tissue section (false coloured for clarity). (**n**–**q**) Increasing magnification of a sectioned carbonized liver shows internal vascularization and resolution of fibrous features (**q**, inset). (**n**–**q)** Scale bars, 250, 50, 10 and 1 μm, respectively.

**Figure 4:**
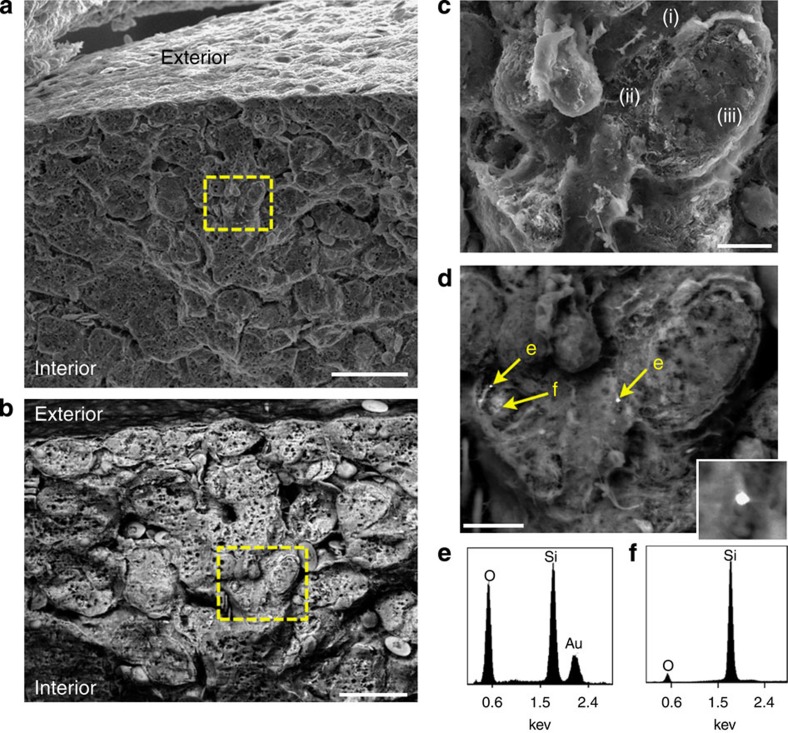
Chemical fingerprinting of gold nanoparticles in the interior of a chicken liver. A mechanically fractured silica bioreplicated and carbonized (c-SBR) chicken liver imaged using secondary electron (SE) (**a**, scale bar, 50 μm) and backscattered electron detection (BSE) (**b**, scale bar, 50 μm) reveals gross morphology of the internal liver. The area within the dashed rectangle in **a** and **b** is expanded and imaged using SE (**c**, scale bar, 5 μm) revealing a fenestrated sinusoid (i), space of disse (ii) and hepatocyte (iii). This same region is imaged using BSE to reveal single 200-nm diameter AuNPs (**d**, scale bar, 5 μm; inset is a magnification of the centre bright spot). The spectrum in **e** was acquired from the centre particle (and representative of spectra obtained from other points denoted as ‘e’) and the spectrum in **f** was acquired from the region ‘f’ denoted in **d**. (**e**,**f**) Show relative intensities from single pixel acquisitions of >1,000 counts.

To investigate shape-preserving transformation of specimens into conductive replicas, we pyrolysed silicified chicken embryo tissues in a tube furnace under both inert (N_2_ or Ar) and reducing environments (5% H_2_ in N_2_) as shown schematically in Fig. 3a. Figure 3b–h show images obtained from a carbonized silica bioreplicated (c-SBR) chicken heart (1,000 °C, 12 h, in Ar) where preservation of the structure is maintained across scales, qualitatively similar to our observations of calcined silicified tissue (Figs 1 and 2). To investigate gross structural changes that may occur from reductive pyrolysis, we subjected two chicken hearts to a side-by-side comparison with or without silicic acid treatment. For the silicified heart, the relative size changes from the hydrated to dehydrated and dehydrated to pyrolysed were minor, with the structure maintaining ~94% of its dehydrated size following pyrolysis (Supplementary Fig. 3). For the untreated heart (that is, no SBR), the size changes were substantial (Supplementary Fig. 3). For any sample, some shrinkage is expected following dehydration. For the untreated heart, we performed a careful sequential dehydration (typically five to seven steps) and subsequent air drying from hexamethyldisilazane (HMDS), which is a common biological specimen dehydration procedure for EM^9^. Here, the overall structure and features of the tissue are maintained and shrinkage (in this case ~28%) is expected upon dehydration^5^. However, the silicified heart displayed much less shrinkage (~7%) and dehydration can be achieved in a single step (air dried from methanol). Further, the differences in size changes between the silicified and untreated heart from the dehydrated state to the pyrolysed state (~6 and ~57%, respectively) were much more substantial. Most importantly, cellular structure was completely lost in the untreated heart (Supplementary Fig. 4) versus the silicified tissues and organs (Figs 1, 2, 3, 4).

Next, we investigated whether electrical conductivity was maintained throughout the tissue by mechanically sectioning a pyrolysed embryo heart. As shown in Fig. 3i–m, the internal structures of the heart—including the large internal spaces of the heart chambers—remained intact (that is, had not collapsed), and high resolution images of individual cells and surfaces could be acquired deep within the heart chamber (~1–2 mm). Importantly, direct imaging of surfaces deep within tissues using EM presents many challenges. Environmental SEM allows internal imaging of biological structures but is currently only amenable to very thin samples of the order of single cells^23^. Otherwise, internal imaging generally requires serial sectioning of an embedded specimen followed by virtual reconstruction or, alternatively, careful dissection, preparation (fixation/dehydration) and metallization of a specimen. With the latter, any further sectioning would necessitate additional surface metallization. Here the intrinsically conductive internal surfaces combined with the dynamic depth of field of an SEM enables imaging deep within internal cavities and allows biomolecular structures to be directly resolved within their 3D context and, if required, subsequent sectioning of the stabilized structure can be achieved manually, mechanically or by FIB without the need for sputter coating or heavy metal staining. FIB/SEM may prove particularly suitable for c-SBR specimens (Supplementary Fig. 5) as a means to shorten FIB processing time, which can take days due to the complexities of sample preparation, milling and image processing^10^. To illustrate simple manual dissection of a c-SBR specimen, Fig. 3n–q shows a sectioned liver with preservation of features down to ~20–30 nm (Fig. 3q, inset). Here, the dense specimen appears to have been sectioned along intrinsic fracture planes (for example, intercellular spaces) revealing a snapshot of internal surface topography, vascular hierarchy and cellular organization that otherwise would be flattened using mechanical methods of sectioning such as microtome or FIB.

### Nanoparticle detection in the interior of an organ

The electrical conductivity of these specimens allows SEM interrogation using high currents (10s of nA) and accelerating voltages (10–30 kV) that could otherwise damage even metal-coated samples (where metal coatings are typically ~10–20 nm thick). This may allow for chemical/elemental analysis using BSE imaging, which requires high current/kV for sufficient contrast. Considering the increasingly widespread interest in metal and other nanoparticle materials for medicine^24^ and derivative studies^25^ (for example, nanoparticle toxicology, biodistribution, tissue/particle interactions), the ability to detect nanoparticles in deep tissue, particularly at low densities with single particle resolution and within the intact 3D architecture of the tissue microenvironment, remains a challenge. Though BSE detection has been occasionally applied to biological materials, examples have required specialized instrumentation (variable pressure/beam deceleration^26^,^27^) or sample preparations (for example, single cells grown on conductive substrates^28^) that are incompatible with normal tissue development.

Thus, we investigated whether SBR carbonization (c-SBR), combined with mechanical sectioning and BSE could detect intravenously injected nanoparticles within tissues. For this experiment, we synthesized 200 nm gold nanoparticles (AuNPs—stabilized with thiolated polyethylene glycol; Supplementary Fig. 6) and injected them into a 16-day-old chicken embryo. NPs were introduced by direct injection into a vein of the chorioallantoic membrane and were allowed to circulate for 1.5 h. Here, it was expected that AuNPs would deposit preferentially within the liver tissue soon after injection due to their relatively large size^29^. After harvesting and preparing the liver (using c-SBR), the tissue was mechanically sectioned across a large lobe to reveal the internal structure (Fig. 4). Figure 4a,b show lower magnification secondary electron (SE) and BSE images of the sectioned tissue that provide complementary views; features such as microvilli and fenestrations are readily identified in SE mode while subsurface structures including cell nuclei are apparent using BSE detection. Focusing in and using a driving current of ~20 nA at 10 kV, BSE revealed highly contrasted, individual AuNPs arrested on the walls of the sinusoid endothelium (Fig. 4d and Supplementary Fig. 6). These particles could be chemically fingerprinted *in situ* from the surrounding background using energy-dispersive X-ray spectroscopy as shown in Fig. 4e,f. BSE detection is essential as the particles were indiscernible from the surrounding tissue using secondary electron (SE) detection (Fig. 4c). This indicates that c-SBR procedures do not appear to detrimentally alter the physical properties of AuNPs, and exemplifies the type of problem that is particularly well suited to be addressed using this approach. Depending on instrumentation, imaging conditions and sufficient Z-contrast with the carbonized specimen, particles that span the size ranges currently investigated for diagnostic and therapeutic applications (10s of nm to ~microns) should be detectable.

## Discussion

The simplicity of the technique, specimen stability, intrinsic conductivity post carbonization and level of resolution spanning six orders of magnitude of magnification (that is, whole embryo to subcellular) distinguishes SBR from all previous bio-preservation methods and should facilitate the examination of soft tissues in their native 3D conformation that were previously difficult or impossible to achieve. As an example, as BSEs are scarcer and emanate from a deeper interaction volume (~1–2 microns^30^) in comparison with SE, the sample subsurface could be resolved non-destructively, revealing the architecture underlying a tissue surface (Supplementary Fig. 7). Our ability to discover and image nano-objects within a biological tissue/organism—finding essentially ‘a needle in a haystack’—while maintaining 3D context is a new capability. While complete tissues and organs have been preserved, immunostained and optically imaged after stabilization within hydrogels and refractive index matching^31^, further EM characterization of such hydrogel-stabilized samples required multiple steps of solvent exchange, epoxy impregnation to provide stability, staining to provide contrast and ultra-microtoming to achieve thin sections. Here stabilization of complete organisms by ultra-thin conformal silica layers forms a mechanically robust, refractory replica allowing transformation to carbon, dissection and EM imaging *ad infinitum* at different scales of magnification and with apparent generalizability to other soft tissues derived from model organisms (for example, mouse; Supplementary Fig. 8). Although intracellular structures can be imaged (Supplementary Fig. 5), resolution of such features currently does not approach methods that use sectioning (for example, serial block-face); however, the size of samples that can be used is only limited by the instrumentation implemented for material processing and imaging (for example, size of pyrolysis furnace, chamber volume of SEM and so on). While detailed interpretation of structures and structural accuracy with this new approach require continuing efforts, our procedure to impart shape-preserving, intrinsic conductivity across all specimen planes could inform further design in instrumentation to optimize the resolution of buried features, which may require higher energy fluxes and more sophisticated aberration correction and deconvolution methods.

## Methods

### Chicken embryo incubation and preparation

*Ex ovo* chicken embryo experiments were conducted under UNM protocol #10-100652-T-HSC, with all embryos used between day 3 and 17 (and as indicated in each experiment). All embryos were handled and euthanized following approved UNM Institutional Animal Care and Use Committee (IACUC) procedures. Fertilized chicken eggs were obtained from East Mountain Hatchery (Edgewood, NM) and placed in an automated incubator (GQF 1500 professional, Savannah, GA) for 72–96 h, humidified (70% RH) and heated (37 °C). Following incubation, the egg shells were sterilized by brief immersion in ethanol and physically cleaned with a paper towel. The egg shells were then scored using a rotary tool and cracked into a medium sterilized weigh boat (VWR). Weigh boats were covered with a square plastic petri dish (VWR) and returned to the incubator until they were killed or time of injection. For particle injections, 0.1 ml of AuNPs (0.25 OD at *λ*=600 nm) were injected via a pulled glass capillary needle into the vein of the chorioallantoic membrane and allowed to circulate for 90 min. Upon removal of embryos from the *ex ovo* egg, tissue was immersed in 3.7% paraformaldehyde in PBS for at least 24 h before silicification. For embryos at day 17 of development, individual organs were dissected from the chicken and fixed individually.

### Silicification of specimens

Following fixation, tissues or whole embryos were silicified by brief rinsing with PBS followed by subsequent immersion in silicification solution in a sealed container at 37 °C for 7–21 days. The silicification solution contains 0.1 M silicic acid derived from hydrolysis of tetramethyl orthosilicate (TMOS) at pH 3 containing 0.154 M NaCl (0.9% saline solution). For example, to make a 100 ml solution, 0.1 ml of 1 N HCl is added to ~98.5 ml of the saline solution. Then, 1.5 ml of TMOS is added to this solution and stirred vigorously (this can be accomplished by shaking in a sealed container) to hydrolyse the TMOS (it will appear dissolved upon hydrolysis) forming principally monosilicic acid Si(OH)_4_. The approximate volume ratio of specimen to solution was kept at or below 1:20 as ratios exceeding 1:10 (specimen:silica solution; v/v) often were observed to induce gelation of the solution (likely due to an increase in solution pH). No obvious difference in gross phenotype was apparent over the course of 3 weeks and gelation of solution (due to silica self-condensation) occurred only if the solution was not refreshed for over 3 weeks. Silica deposition upon specimens was apparent after a few days of immersion in the silicic acid solution by a change in colour of the specimen from pink/brown to white. Following silicification, SBR tissues were rinsed in H_2_O (pH 3), 1:1 water/methanol and finally dried in air from 100% methanol.

### Dehydration of non-silicified tissue

The non-silicified heart tissue shown in Supplementary Fig. 3 was fixed overnight in 3.7 volume% formaldehyde in PBS solution, dehydrated by using 20-min sequential washes (33% ethanol (EtOH) in H_2_O; 50% EtOH; 66% EtOH; 2 × 100% EtOH; 50% EtOH in HMDS; 100% HMDS) and allowed to dry in air for 16 h.

### Calcination and pyrolysis of samples

Silicified samples were calcined by placing them in a covered (but not air tight) pyrex dish and treating for 12–16 h in an oven (Fisher Scientific, Model #495A) at 500 °C under ambient atmospheric conditions. Ramp temperature was controlled at 1 °C per min, however, cooling rate was uncontrolled.

For pyrolysis, silicified and non-silicified samples were placed uncovered in a ceramic combustion boat (~20 × 75 mm W × L alumina or porcelain) and heated to either 800 or 1,000 °C in a quartz tube (25 mm OD; 20 mm ID) inserted in a tube furnace (Lindberg/Blue Model #TF55035A) under constant gas flow (N_2_, Ar or 5% H_2_ in N_2_); the heating rate was 5 °C per min and final temperature was held for 12 h. We found that an 800 °C holding temperature was sufficient for carbonization of samples and higher temperatures were not required.

### Scanning electron microscopy/energy-dispersive spectroscopy

SEM images were recorded using an FEI Quanta series SEM located at The University of New Mexico. This instrument was equipped with an energy-dispersive X-ray spectroscopy from EDAX, which was used in single pixel mode for elemental identification. For SEM images shown in Fig. 1, samples were sputter-coated with Au/Pd. In addition, FIB milling shown in Supplementary Fig. 5 was performed on this instrument.

### Synthesis of 200 nm gold nanoparticles

Gold nanoparticles (AuNPs) were synthesized according to literature^32^. Briefly, 20 ml of HAuCl_4_ (0.1 mg ml^−1^) was titrated against trisodium citrate (10 mg ml^−1^) at 90 °C. Two hundred nanometre particles are obtained with ~0.35 ml of trisodium citrate. After heating, the mixture was rapidly quenched in an ice bath, and the particles were washed with centrifugation (500 r.c.f., 5 min). After resuspension in DI water, citrate capping was immediately exchanged by drop-wise addition of PEG-thiol in ethanol (MW 5000, 2 mg ml^−1^). The mixture was stirred for 24 h, and particles were purified again by two cycles of centrifugation. The final product was dispersed in PBS at~0.25 OD (*λ*=600 nm).

## Author contributions

J.L.T. and B.K. conceived the study; J.L.T., C.J.B. and B.K. designed the research; J.L.T., Y.-S.L., S.S.C., Y.H.A., E.N.C. and B.K. carried out the experiments; J.L.T., C.J.B. and B.K. analysed the data; J.L.T. and B.K. wrote the paper; all the authors commented on and edited the final version of the paper.

## Additional information

**How to cite this article**: Townson, J. L. *et al.* Synthetic fossilization of soft biological tissues and their shape-preserving transformation into silica or electron-conductive replicas. *Nat. Commun.* 5:5665 doi: 10.1038/ncomms6665 (2014).

## Supplementary Material

Supplementary Figures Supplementary Figures 1-8
