# Supplementary material for: Synthetic fossilization of soft biological tissues and their shape-preserving transformation into silica or electron-conductive replicas
Source: Nat Commun. 2014 Dec 8;5:5665. doi: 10.1038/ncomms6665 (PMC4268709; doi:10.1038/ncomms6665)
Supplement: Supplementary Figures — 1-8 [file ncomms6665-s1.pdf]

Supplementary Figures

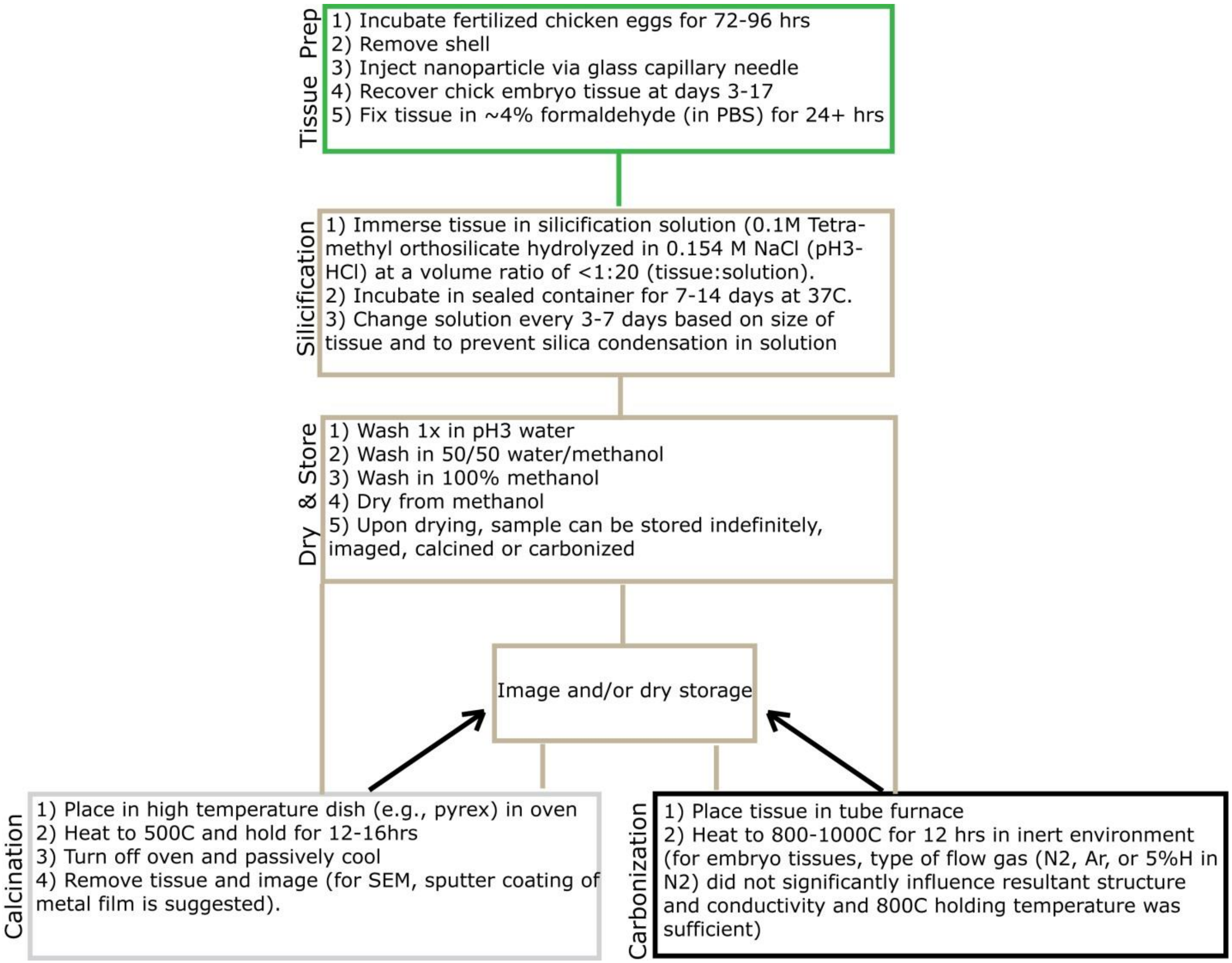

Supplementary Figure 1. Flow chart of SBR process for chicken embryo specimen.

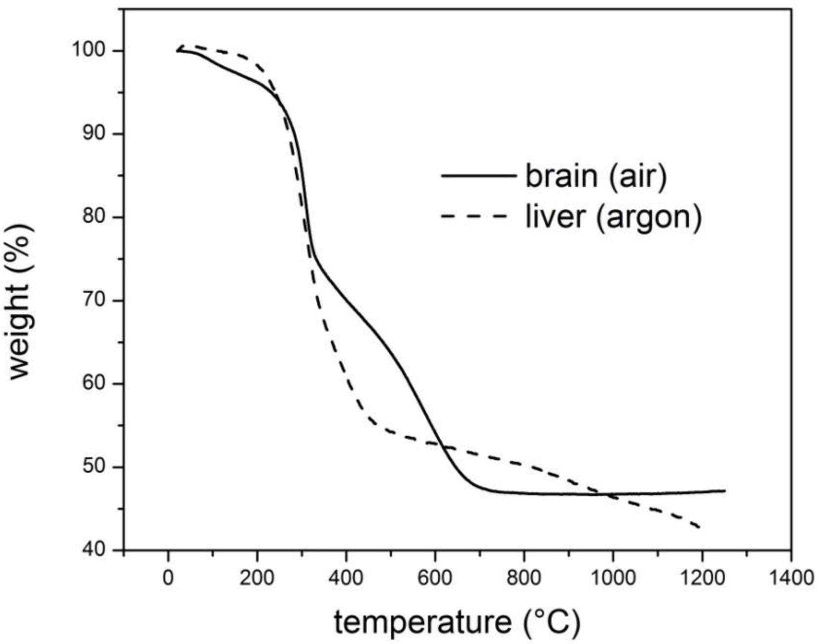

Supplementary Figure 2. Thermogravimetric analysis (TGA) curve of a silicified embryo tissue produced by the SBR process, recorded under flowing air and argon (50 ml min^{-1}).

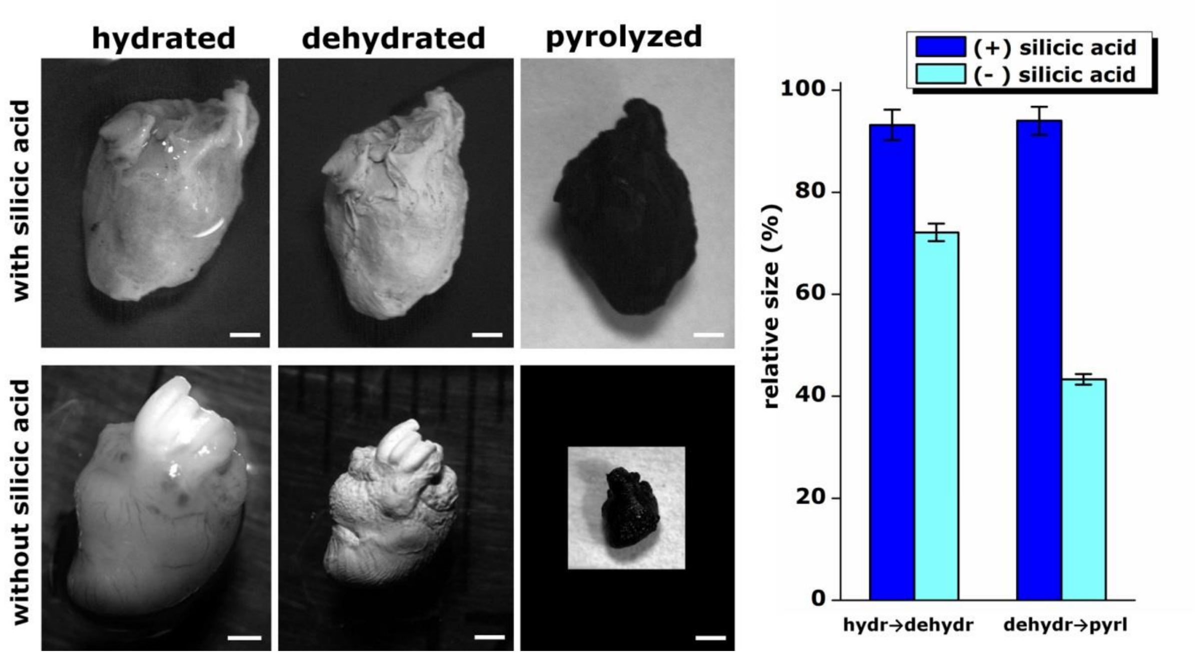

Supplementary Figure 3. Size comparison of dehydration and pyrolysis of a silicified versus non-silicified heart. The graph shows the relative size changes following hydration to dehydration and dehydration to pyrolysis based on comparison of multiple line measurements from these images. All scale bars are 1 mm.

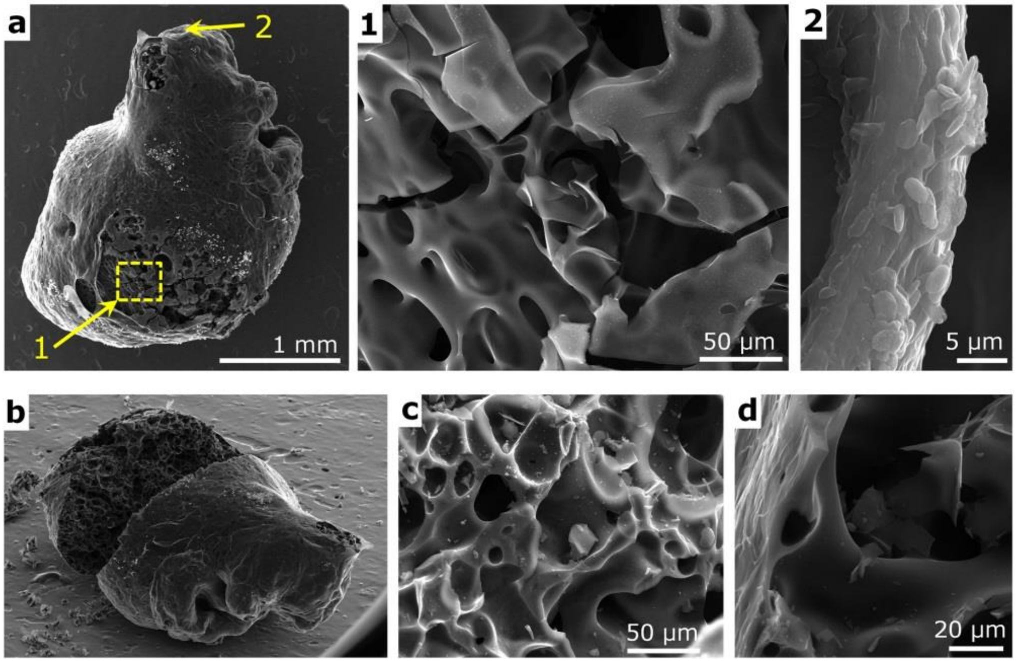

Supplementary Figure 4. (a) SEM images of a non-silicified pyrolyzed heart and magnified regions in panels 1 and 2. Panel 1 shows total loss of cellular and extracellular features. Interestingly, panel 2 shows what appear to be RBC-like shapes. (b, c, & d). Interior features of the non-silicified pyrolyzed heart show no discernable biological structures.

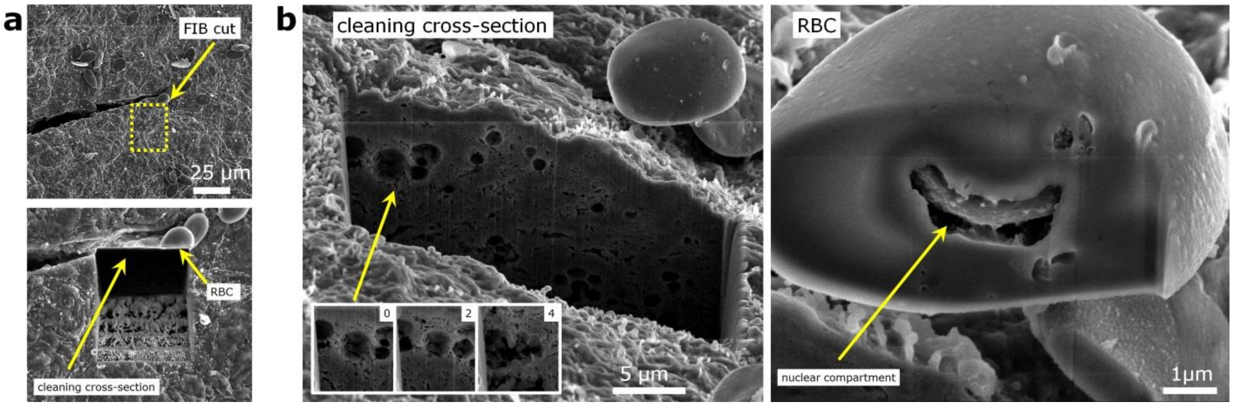

Supplementary Figure 5. FIB milling of a c-SBR chicken liver. (a) Cleaning cross-section of surface of the liver and a red blood cell (RBC) using 15 nA (~ 8 minutes milling time). (b, left panel) The face of the cleaning cross-section shows a sinusoidal space that is milled approximately 4 microns further (inset, numbers indicate distance of milling in microns). (b, right panel) Milled chick RBC reveals the nuclear compartment (compared to mammalian RBCs, chick RBCs are nucleated).

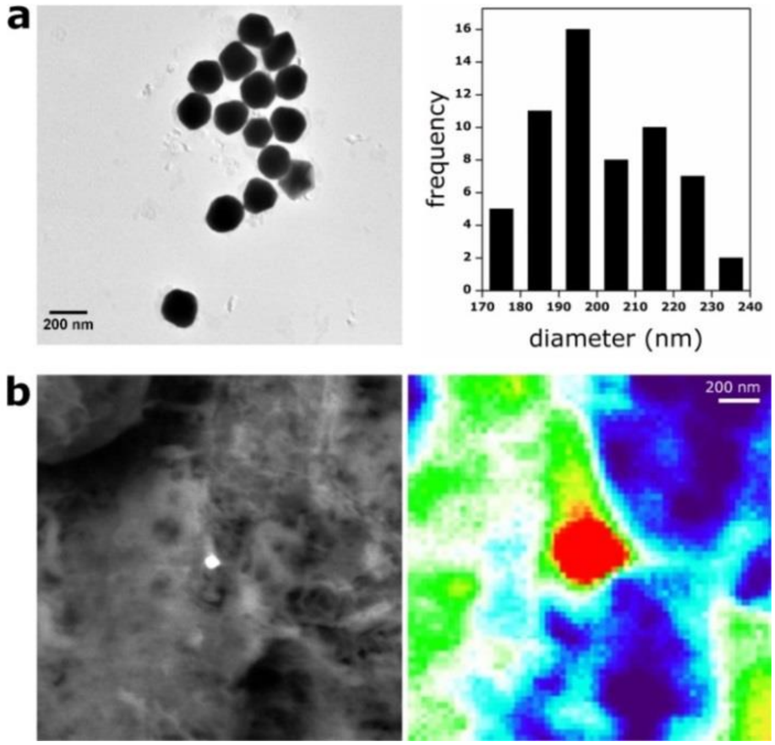

Supplementary Figure 6. (a) TEM images of and particle size histogram of AuNPs prior to injection. (b) SEM-BSE image of a single particle shown in Figure 3 and an intensity map (right panel) to determine size. The red region indicates the area of detector saturation and is ~325 nm in diameter indicating detection of a single, overexposed AuNP corresponding to the size of the injected particles.

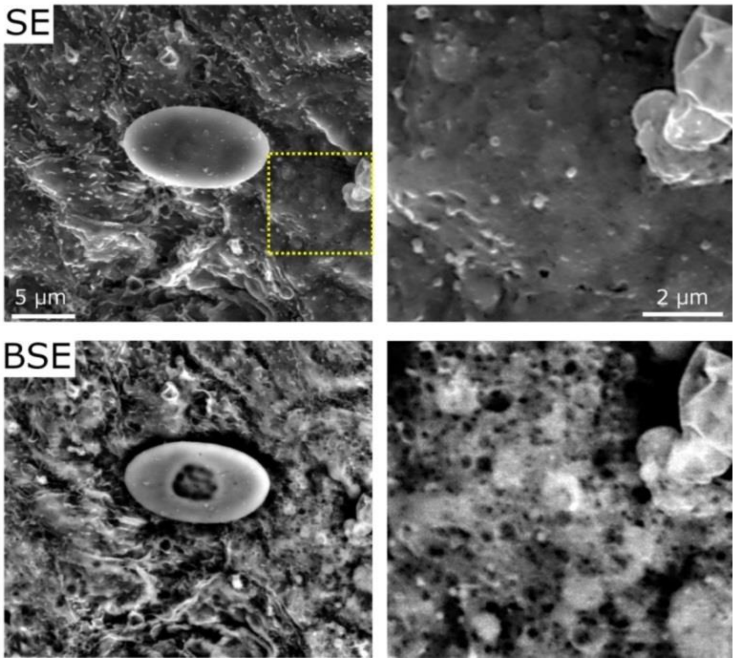

Supplementary Figure 7. Comparison of SE and BSE imaging of c-SBR liver tissue reveals sub-surface intra and extracellular architecture. The dotted rectangle (top left) is magnified in the right panels. The RBC nuclear region show void spaces (dark regions) in the BSE image (bottom left) which indicates compacted nuclear material as verified using FIB (see Supplementary Figure 5).

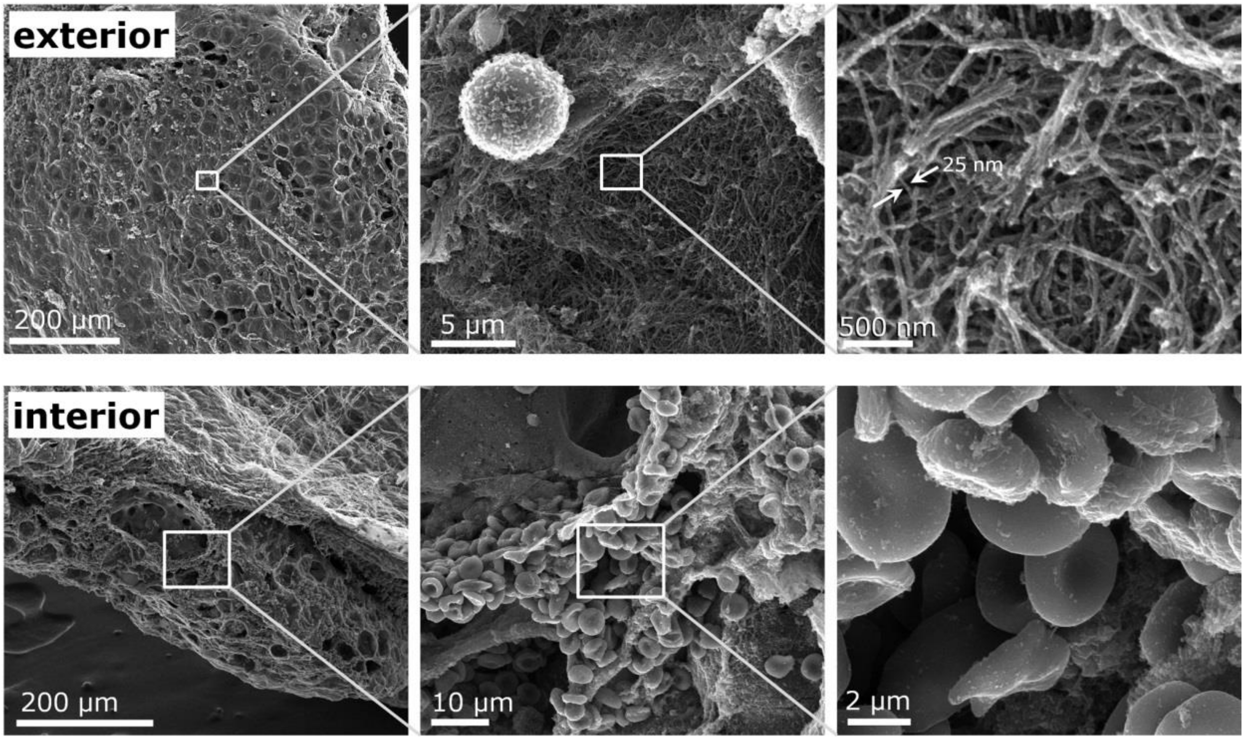

Supplemental Figure 8. Increasing magnification (from left to right) of a carbonized mouse spleen shows the fibrous surface of the exterior capsule (top panels) and the red blood cell rich internal sinusoids of the red pulp (lower panels).
